# Sugar-Sweetened Beverages Consumption in a Multi-Ethnic Population of Young Men and Association with Sociodemographic Characteristics and Obesity

**DOI:** 10.3390/ijerph20064861

**Published:** 2023-03-09

**Authors:** Jozaa Z. AlTamimi, Naseem M. Alshwaiyat, Hana Alkhalidy, Nora M. AlKehayez, Reham I. Alagal, Reem A. Alsaikan, Malak A. Alsemari, Mona N. BinMowyna, Nora A. AlFaris

**Affiliations:** 1 Department of Physical Sports Sciences, College of Education, Princess Nourah bint Abdulrahman University, P.O. Box 84428, Riyadh 11671, Saudi Arabia; jzaltamimi@pnu.edu.sa (J.Z.A.); nmalkehayez@pnu.edu.sa (N.M.A.); 2 School of Nutrition and Dietetics, Faculty of Health Sciences, Gong Badak Campus, Universiti Sultan Zainal Abidin, Kuala Nerus 21300, Terengganu, Malaysia; sh_naseem@yahoo.com; 3 Department of Nutrition and Food Technology, Faculty of Agriculture, Jordan University of Science and Technology, Irbid 22110, Jordan; haalkhalidy@just.edu.jo; 4 Department of Health Sciences, College of Health and Rehabilitation Sciences, Princess Nourah bint Abdulrahman University, P.O. Box 84428, Riyadh 11671, Saudi Arabia; rialagal@pnu.edu.sa (R.I.A.); raalsakan@pnu.edu.sa (R.A.A.); 5 Department of Medical Imaging—MRI, King Abdullah bin Abdulaziz University Hospital (KAAUH), Princess Nourah bint Abdulrahman University, Riyadh 11671, Saudi Arabia; maalsemari@kaauh.edu.sa; 6 College of Science and Humanities, Shaqra University, Shaqra 11911, Saudi Arabia; m.mwena@su.edu.sa

**Keywords:** sugar-sweetened beverages, multi-ethnic, young men, sociodemographic, obesity

## Abstract

Sugar-sweetened beverages are frequently consumed among adults and are linked with the incidence of obesity. We aimed to determine rates of weekly and daily sugar-sweetened beverage intake in a multi-ethnic population of young men and their association with sociodemographic characteristics and obesity. This cross-sectional study included 3600 young men who lived in Riyadh, KSA. Participants’ sociodemographic characteristics and frequency of sugar-sweetened beverage consumption were gathered through personal interviews. The outcome variables in this study are based on the weekly and daily consumption of sugar-sweetened beverages. Weight and height were measured following standard protocols. The rates of weekly and daily sugar-sweetened beverage intake by participants were 93.6% and 40.8%, respectively. Nationality was a predictor of weekly and daily consumption of sugar-sweetened beverages. The highest rates of weekly (99.5%) and daily (63.9%) consumption were observed in subjects from the Philippines and Yemen, respectively, while Bangladeshi subjects had the lowest rates of weekly (76.9%) and daily (6.9%) consumption. Obesity was another predictor of sugar-sweetened beverage consumption. Obese participants had a significantly higher odds ratio of weekly sugar-sweetened beverage consumption than non-obese subjects (OR = 4.53, *p* = 0.037). In conclusion, sugar-sweetened beverage consumption was relatively high and our results support an association between the consumption of sugar-sweetened beverages and certain sociodemographic variables and obesity.

## 1. Introduction

Sugar-sweetened beverages (SSBs) are considered among the leading energy-dense foods in terms of consumption globally [1]. They are defined as beverages prepared with added sugar [2]. Unfortunately, SSBs are a main dietary source of added sugar intake [3]. They are rich in calories and poor in nutrients which may negatively affect overall diet quality [4]. Surplus sugar consumption seems to have become a serious public health issue worldwide [5]. Therefore, the WHO advises keeping consumed added sugar lower than 10% of daily calories [5]. Current evidence indicates that frequent SSB intake is linked to gaining weight and could cause obesity and obesity-related diseases such as type 2 diabetes and cardiovascular disease [6,7]. Nevertheless, declining SSB consumption may lower obesity incidence and many other interrelated chronic diseases [8]. Consumption of SSBs is highly prevalent among adults, especially young men [9,10]. Therefore, understanding patterns of SSB consumption and any associated sociodemographic characteristics is necessary for developing effective public health strategies to lower the consumption of foods with added sugars, especially SSBs [10].

Obesity is a serious health issue in the Kingdom of Saudi Arabia (KSA) and is concurrent with unhealthy lifestyles, including sedentary behaviors, low-quality diet, and high intake of SSBs [11,12,13]. In 2019, the Saudi Ministry of Health implemented a nationally representative health survey targeting adults aged fifteen years or older [14]. They found that the prevalence of those that were overweight and obese were 38% and 20%, respectively. Males were more likely to be overweight (43%) than females (33%), whereas females were more likely to be obese (21%) than males (19%). Moreover, the prevalence of obesity rises with age from 10% in the age group 18–29 years to 29% in the age group 70–79 years, before dropping to 22% in the respondents aged 80 years or over [14]. Abnormal waist circumference, defined as a waist circumference of more than 80 cm in women and more than 94 cm in men, was reported among 30% of respondents (34% in females vs. 27% in males). There was a steady increase in abnormal waist circumference with age, from 23% in the age group 15–29 years to 58% in the age group 80 years or over. The majority of respondents (91%) have an abnormal waist–hip ratio defined as a waist–hip ratio equal to or more than 0.85 and 0.9 in women and men, respectively [14]. Hypertension was observed among 14% of respondents (12% in females vs. 15% in males). The prevalence of hypertension dramatically increases with age from 6% in the age group 15–29 years to 56% in the age group 80 years or over [14]. The prevalence of respondents with impaired glucose tolerance (random glucose levels ≥ 7.8 and <11.1 mmol/L) was 11%, while the prevalence of diabetes mellitus (random glucose levels ≥ 11.1 mmol/L) was 4%. The prevalence of diabetes mellitus increases with age from 4% in the age group 15–29 years to 7% in the age group 80 years or over [14]. Hypercholesterolemia (total cholesterol ≥ 5 mmol/L) was reported among 43% of respondents. The rate of respondents with hypercholesterolemia increases dramatically with age from 39% in the age group 15–29 years to 68% in the age group 80 years or over [14]. Furthermore, the ten leading causes of mortality in 2019 in KSA were ischemic heart disease, road injuries, stroke, chronic kidney disease, lower respiratory infections, falls, cirrhosis, diabetes, other unintentional causes, and chronic obstructive pulmonary disease (COPD) [15]. The Kingdom leads the Middle East region in oil production and has a rapidly expanding economy. Not surprisingly, KSA attracts employees from various countries around the world. Around 50% of the country’s workforce and about 90% of jobs in the private business sector in KSA are occupied by expatriates [16]. Approximately, 30% of the inhabitants in KSA are not Saudi citizens, and most of them are males [17]. Studying the disparities in diet patterns and their relationship to illness incidence in diverse populations is a fascinating area of research once the ethnic origins of this expatriate population are considered more closely. Therefore, the present study aimed to assess the prevalence of weekly and daily SSB intake in a multi-ethnic sample of young men living in KSA and examine the association between the consumption of SSBs and related sociodemographic factors and obesity. Our results will be valuable for policy-makers in the health care system in KSA, especially when developing strategies to reduce the consumption of SSBs for public and high-risk subgroups.

## 2. Methods

### 2.1. Research Design and Subjects

Current data are from a cross-sectional research project called ROAD-KSA which was carried out from February to June 2019 and targeted young and middle-aged men [18,19,20,21,22,23,24,25]. Study subjects were randomly selected from public sites in Riyadh using a stratified clustered sampling method. The eligibility criteria included young men (20 to 35 years), who lived in Riyadh, were physically fit, and were citizens of one of the following countries: KSA, Egypt, Yemen, Syria, Jordan, Sudan, Turkey, Pakistan, Afghanistan, India, Bangladesh, and the Philippines. Per the Helsinki Declaration, participants signed informed consent forms. This work was ethically cleared by the research ethics committee/Princess Nourah bint Abdulrahman University.

### 2.2. Sociodemographic Characteristics

Personal interviews were adopted to find the sociodemographic characteristics of subjects. Collected variables include nationality, age, residency period, household type, marital status, educational level, and monthly income.

### 2.3. Measurements

Weight and height were measured by a qualified research team. Weight was measured to the nearest 0.1 kg by digital weight balance with the participants wearing light clothing and being barefoot. In the same way, height was measured to the nearest 0.1 cm by a portable stadiometer in a standing position and also barefoot. Body mass index (BMI) was derived by dividing weight in kilograms by height in squared meters. Obesity is defined as BMI ≥ 30 [26].

### 2.4. Sugar-Sweetened Beverage Consumption

A valid and reliable questionnaire was used to determine the frequency of SSB consumption. An impartial judgment from five nutrition research specialists examined the questionnaire’s face validity. The questionnaire’s reliability was measured using a test–retest pilot study with a two-week delay. Personal interviews were adopted to gather data. This study defines SSBs as manufactured drinks that contain added simple carbohydrates such as fructose and sucrose and does not include milk, tea, coffee, or alcohol [27]. Therefore, SSBs are categorized into the following products: regular soda, fruit drinks, and energy drinks. Regular soda is typically defined as a carbonated drink that is sweetened with simple carbohydrates. Fruit drinks involve fruit beverages that contain added simple carbohydrates but do not comprise 100% natural fruit juices. Energy drinks refer to artificial drinks with a high level of a stimulant ingredient (usually caffeine) and added sugar. The frequency of SSB consumption was assessed by questioning the subjects about how many servings (about 12 fluid-ounce units) of each SSB product they consumed weekly or daily in the previous twelve months. The prevalence of weekly and daily intake of overall SSBs and individual products of SSBs were calculated according to the following definitions. Weekly and daily consumption were specified as consuming a minimum of one serving in a typical week or day, respectively [28].

### 2.5. Statistical Analysis

For data analysis, IBM SPSS Statistics for Windows (version 26. Armonk, New York, NY, USA, 2019) was used. Two binary outcome variables were used in this study: the weekly and daily consumption of SSBs. The analysis of categorical variables was handled using the Chi-square test, which was then reported as frequencies and percentages. To identify the variables connected to weekly and daily SSB consumption, a multivariate logistic regression analysis was run by adjusting for studied sociodemographic variables and obesity. All of the *p*-values were calculated using two-tailed testing. Statistical significance was regarded only when *p*-values were lower than 0.05.

## 3. Results

This research involved 3600 participants. The prevalence of weekly SSB consumption among study participants stratified by sociodemographic characteristics and obesity is displayed in Table 1. For the complete study sample, the prevalence of weekly SSB consumption was 93.6%, whilst it was 86.9% for regular soda, 60.0% for fruit drinks, and 41.4% for energy drinks. Stratifying subjects based on nationality shows significant differences (*p* ˂ 0.001) in rates of weekly SSB intake. The highest rate of SSB consumption per week was detected in participants from the Philippines (99.5%), while the lowest prevalence was detected in Bangladeshi subjects (76.9%). Participants who had resided for six years or more in KSA had a significantly higher prevalence of weekly SSB intake (96.2%) than those with five years or less of a residency period (91.9%). Interestingly, single participants had a significantly greater prevalence of weekly SSB intake (95.5%) than married participants (91.4%). Obese participants had a significantly higher rate of weekly SSB intake (99.1%) than non-obese participants (93.2%).

The prevalence of daily SSB intake stratified by sociodemographic characteristics and obesity is presented in Table 2. For the complete study sample, the rate of daily SSB intake was 40.8%, whilst it was 14.9% for regular soda, 3.9% for fruit drinks, and 1.8% for energy drinks. Stratifying participants based on nationality shows significant differences (*p* ˂ 0.001) in daily SSB consumption rates. Yemeni subjects had the highest rates of daily intake of SSBs (63.9%), regular soda (32.5%), fruit drinks (11.6%), and energy drinks (6.3%). Contrarily, Bangladeshi subjects had the lowermost rates of daily intake of SSBs (6.9%), regular soda (2.0%), and fruit drinks (0.0%). Participants who lived within family households had a significantly greater daily SSB consumption rate (45.7%) than those who lived away from their families (39.6%). Single subjects had significantly greater daily intake rates of SSBs (44.3%), regular soda (17.1%), and fruit drinks (4.7%) than married participants (36.8%, 12.5%, and 2.9%, respectively). Surprisingly, participants with high education levels had a significantly higher daily SSB consumption rate (48.8%) than those with low education levels (36.2%). Obese subjects had higher daily consumption rates of SSBs, regular soda, fruit drinks, and energy drinks (45.9%, 23.0%, 4.1%, and 2.7%, respectively) than non-obese subjects (40.4%, 14.4%, 3.9%, and 1.7%, respectively). However, the difference was only significant in the case of regular soda (*p* ˂ 0.05).

The likelihoods of weekly and daily SSB intake for sociodemographic characteristics and obesity are displayed in Table 3. Nationality was a predictor of SSB intake per week or day. Compared with Bangladeshi subjects, those from other countries had a significantly higher likelihood of weekly SSB intake (odds ratio [OR] ranged from 2.15 to 44.98, *p* ˂ 0.05). In the same way, compared with participants from Bangladesh, participants from other countries (except Sudan) had a significantly higher likelihood of daily SSB intake (OR ranging from 6.69 to 29.94, *p* ˂ 0.001). Advance in age was significantly associated with a lower likelihood of daily SSB consumption (OR = 0.96, *p* = 0.001). Moreover, subjects who resided for six years or more in KSA had a significantly higher likelihood of weekly SSB consumption than those with five years or less of a residency period (OR = 1.99, *p* ˂ 0.001). Married participants had a significantly lower likelihood of weekly SSB consumption (OR = 0.52, *p* ˂ 0.001) than single subjects. Participants with a high monthly income had a significantly lower likelihood of weekly (OR = 0.37, *p* ˂ 0.001) and daily (OR = 0.58, *p* ˂ 0.001) SSB consumption than those with a low monthly income. Lastly, obese participants had a significantly higher likelihood of weekly SSB consumption than non-obese participants (OR = 4.53, *p* = 0.037).

## 4. Discussion

The current study looked at the prevalence of SSB consumption on a weekly and daily basis in a multi-ethnic sample of young men from KSA. Most participants (93.6%) were weekly SSB consumers, and nearly two-fifths of the participants (40.8%) were daily consumers of SSBs. Several studies have examined SSB consumption rates in adults. A recent nationally representative study from KSA found that 71.2% of adults were weekly consumers of SSBs, while 35.5% of adults were daily consumers. Furthermore, 80.2% of young adults aged 25 to 34 years were weekly SSB consumers [28]. Another study reported that 60% of young Jordanian adults were daily SSB consumers [29]. A representative study observed that 47.3% and 13.6% of Australian adults had weekly and daily SSB consumption, respectively. The prevalence of weekly and daily SSB consumption among young adults (18–30 years) was 67.1% and 17.2, respectively [10]. Another report from the United Kingdom found that daily SSB consumption was observed in 20.4% of adults [9]. According to a Scandinavian survey, 41% of men in Norway regularly consume SSBs [30]. Data from five nationally representative surveys (NHANES 1999–2000 to 2007–2008) showed that the SSB consumption rate in young American adults (20–34 years) ranged between 73% and 78%, whilst the prevalence of SSB heavy consumption among them ranged between 20% and 29% [31]. A Malaysian study reported that the rate of daily SSB intake in young adults was 89.3% [32].

Our findings revealed significant variations in rates of SSB use among participants from various countries. This result is in harmony with results from several earlier studies that reported a significant variation in SSB intake among adults from different countries, geographic regions, or ethnicity [31,33,34]. A study comparing SSB consumption across 187 countries distributed into 21 world geographical regions found substantial variability in SSB consumption. The highest and lowest regional intake levels varied almost tenfold. The Caribbean had the greatest SSB intake, while East Asia had the lowest consumption [33]. Additionally, SSB and SSB subtype consumption by adults varied by region of residence in the USA. Rates of daily SSB consumption in the Northeast, South, West, and Midwest were 68.4%, 66.7%, 61.2%, and 58.8%, respectively. Compared with adults in the South region, adults in the Northeast region had a higher likelihood of daily SSB intake (OR = 1.13), but adults in the Midwest region (OR = 0.70) and the West region (OR = 0.78) had a lower likelihood of daily SSB intake [34]. In another report from the USA, SSB consumption differs by ethnicity. Black and Hispanic young adults had a higher likelihood of drinking SSBs than their White counterparts [31]. The reasons for differences in the rates of SSB intake based on nationality are not completely resolute. However, the possible elucidations could include variations in the environmental exposure to SSBs that the adults experience in their home countries in earlier life periods regarding SSB availability and accessibility [35,36]. Another reason could be the variations in their reception and response to SSB advertising in the host country due to variations in language and cultural norms [37]. In the current study, Bangladeshi and Sudanese participants had relatively the lowest rates of daily SSB intake. In KSA, most residents from Bangladesh and Sudan are less educated, come from rural communities, and have manual-type occupations in the services, construction, or agricultural sectors. This could make them less exposed to SSB consumption and be reflected in their dietary behavior. However, differences in SSB intake rates by nationality should be considered when interventions to reduce SSB intake are planned [38].

Discrepancies in SSB intake rates in adults by sociodemographic characteristics have been well recognized in the literature [39]. Our results were consistent with results from former studies that described a greater likelihood of SSB intake among subjects of a younger age [10,28,32,39,40,41]. The current study found significant associations between weekly consumption of SSBs and age, residency duration, marital status, and monthly income. We found that a longer residency duration in KSA was associated with a higher likelihood of weekly SSB consumption. The development experienced in this country and how a modified lifestyle has affected adults’ choices may be to blame for this [42]. As immigrants live in a new country for a longer time, their health will worsen. Health problems may be driven by cultural influences, social and economic changes, as well as adjustments to eating habits created by migration [43]. Another finding was that single subjects had a higher likelihood of SSB intake than their married counterparts. Single individuals frequently consume fast foods, which is linked to a higher SSB intake [28,41]. Our results agreed with past studies that noticed that a greater likelihood of SSB intake was associated with lower monthly income [9,31,32]. Many people do not have continuous access to healthy food due to their economic status. Budget-friendly food choices such as fast food and SSBs are usually low in essential nutrients and high in calories. People with low income are at higher risk of nutritional deficiencies and health problems such as obesity, diabetes, and cardiovascular disease. Therefore, addressing disparities in food access for different groups of the community, especially those with a low income, is a crucial element to achieve food justice and promote public health. Food justice aims to guarantee that everyone has access to nutritious, affordable, and culturally acceptable food [44].

Remarkably, our results confirmed the relationship between weekly SSB intake and obesity. Obesity is an expensive illness with elevated rates of morbidity and mortality that affect many adults [45,46]. Serval studies linked the consumption of SSBs to obesity and other obesity-associated diseases [47,48,49]. Several mechanisms were proposed to explain how SSB consumption can result in obesity. The calorie intake of adults is significantly increased by the consumption of SSBs, which shifts the energy balance toward positive [50]. According to a recent study, young Jordanian adults consumed an average of 481 kcal of total daily calories from SSBs [29]. Furthermore, when opposed to consuming calories from solid foods with high fiber content, the fluid calories from SSBs promote more hunger sense, which could cause an excessive intake of calories [51]. Another explanation is higher hepatic lipogenesis due to consuming high fructose from SSBs, which affects insulin secretion and leads to insulin resistance and excess fat deposition [52].

At the community level, SSB intake should be reduced through various environmental and regulatory changes [27]. It may be possible to reduce SSB consumption by limiting their availability in community areas and lower exposure to SSB advertising in mass media [10]. On the other hand, general attempts to limit SSB intake may be helped by increasing public awareness regarding the negative health effects of SSBs through public health education campaigns [53]. Governments have been urged to take action by international public health organizations due to the extensive and rapid rise in SSB consumption. One effective policy to limit SSB intake is forcing taxes on SSB prices [54,55]. Furthermore, strategies that promote using energy-free liquids like water in place of SSBs should be taken into account [55]. The health sector transformation program that is included in the Vision 2030 program for KSA focuses on boosting public health and disease prevention through programs that target different members of the community in the country, including citizens and foreign residents. The key aspect of this program includes legislation that limits access to unhealthy foods such as SSBs by applying taxes and educational programs implemented by various governmental bodies such as the Ministry of Health and universities to promote healthy eating and limit unhealthy foods such as SSBs [28,56].

A few limitations can be viewed in this study. Causality could not be inferred from the significant correlations due to the cross-sectional design. Another problem was relying on participants’ memories while collecting consumption data using a self-reported frequency technique. This method may yield an underestimated SSB intake compared to a 24-h recall dietary method. Because SSBs are defined differently in different studies, comparing our findings with those of earlier research might be difficult. Lastly, our study did not account for daily energy consumption to examine the link between SSB intake and obesity. Nevertheless, the current study offers insightful information on the prevalence of SSB intake and related factors.

## 5. Conclusions

This study found that the prevalence of SSB intake in young men from KSA was comparatively high. The findings revealed that nationality was a predictor of SSB intake per week or day. The results also support a link between obesity and some sociodemographic characteristics and SSB consumption.

## Figures and Tables

**Table 1:** Prevalence of weekly sugar-sweetened beverage (SSB) consumption among study participants (*n* = 3600) stratified by sociodemographic characteristics and obesity.

Variables	Total N	Total SSBs		Regular Soda		Fruit Drinks		Energy Drinks	
		Yes N (%)	No N (%)	Yes N (%)	No N (%)	Yes N (%)	No N (%)	Yes N (%)	No N (%)
All Participants	3600	3368 (93.6%)	232 (6.4%)	3130 (86.9%)	470 (13.1%)	2159 (60.0%)	1441 (40.0%)	1491 (41.4%)	2109 (58.6%)
Nationality									
Saudi Egyptian Yemeni Syrian Jordanian Sudanese Turkish Pakistani Afghan Indian Bangladeshi Filipino *p*-value *	289 289 335 293 280 276 203 306 303 297 350 379	280 (96.9%) 286 (99.0%) 331 (98.8%) 276 (94.2%) 234 (83.6%) 243 (88.0%) 197 (97.0%) 294 (96.1%) 293 (96.7%) 288 (97.0%) 269 (76.9%) 377 (99.5%) **˂0.001**	9 (3.1%) 3 (1.0%) 4 (1.2%) 17 (5.8%) 46 (16.4%) 33 (12.0%) 6 (3.0%) 12 (3.9%) 10 (3.3%) 9 (3.0%) 81 (23.1%) 2 (0.5%)	257 (88.9%) 270 (93.4%) 299 (89.3%) 249 (85.0%) 206 (73.6%) 232 (84.1%) 184 (90.6%) 283 (92.5%) 279 (92.1%) 271 (91.2%) 249 (71.1%) 351 (92.6%) **˂0.001**	32 (11.1%) 19 (6.6%) 36 (10.7%) 44 (15.0%) 74 (26.4%) 44 (15.9%) 19 (9.4%) 23 (7.5%) 24 (7.9%) 26 (8.8%) 101 (28.9%) 28 (7.4%)	232 (80.3%) 216 (74.7%) 286 (85.4%) 218 (74.4%) 206 (73.6%) 56 (20.3%) 107 (52.7%) 147 (48.0%) 145 (47.9%) 138 (46.5%) 78 (22.3%) 330 (87.1%) **˂0.001**	57 (19.7%) 73 (25.3%) 49 (14.6%) 75 (25.6%) 74 (26.4%) 220 (79.7%) 96 (47.3%) 159 (52.0%) 158 (52.1%) 159 (53.5%) 272 (77.7%) 49 (12.9%)	123 (42.6%) 110 (38.1%) 168 (50.1%) 153 (52.2%) 103 (36.8%) 80 (29.0%) 92 (45.3%) 71 (23.2%) 134 (44.2%) 104 (35.0%) 106 (30.3%) 247 (65.2%) **˂0.001**	166 (57.4%) 179 (61.9%) 167 (49.9%) 140 (47.8%) 177 (63.2%) 196 (71.0%) 111 (54.7%) 235 (76.8%) 169 (55.8%) 193 (65.0%) 244 (69.7%) 132 (34.8%)
Residency Period in KSA									
1–5 years 6 years or more *p*-value *	2198 1402	2019 (91.9%) 1349 (96.2%) **˂0.001**	179 (8.1%) 53 (3.8%)	1985 (86.2%) 1235 (88.1%) 0.104	303 (13.8%) 167 (11.9%)	1250 (56.9%) 909 (64.8%) **˂0.001**	948 (43.1%) 493 (35.2%)	928 (42.2%) 563 (40.2%) 0.220	1270 (57.8%) 839 (59.8%)
Household Type									
Non-family household Family household *p*-value *	2920 680	2726 (93.4%) 642 (94.4%) 0.313	194 (6.6%) 38 (5.6%)	2559 (87.6%) 571 (84.0%) **0.011**	361 (12.4%) 109 (16.0%)	1651 (56.5%) 508 (74.7%) **˂0.001**	1269 (43.5%) 172 (25.3%)	1225 (42.0%) 266 (39.1%) 0.177	1695 (58.0%) 414 (60.9%)
Marital Status									
Single Married *p*-value *	1919 1681	1832 (95.5%) 1536 (91.4%) **˂0.001**	87 (4.5%) 145 (8.6%)	1705 (88.8%) 1425 (84.8%) **˂0.001**	214 (11.2%) 256 (15.2%)	1215 (63.3%) 944 (56.2%) **˂0.001**	704 (36.7%) 737 (43.8%)	873 (45.5%) 618 (36.8%) **˂0.001**	1046 (54.5%) 1063 (63.2%)
Education Level									
Low (high school or less) High (college or more) *p*-value *	2284 1316	2123 (93.0%) 1245 (94.6%) 0.052	161 (7.0%) 71 (5.4%)	1987 (87.0%) 1143 (86.9%) 0.903	297 (13.0%) 173 (13.1%)	1147 (50.2%) 1012 (76.9%) **˂0.001**	1137 (49.8%) 304 (23.1%)	886 (38.8%) 605 (46.0%) **˂0.001**	1398 (61.2%) 711 (54.0%)
Monthly Income									
Low (˂1000 USD) High (≥1000 USD) *p*-value *	2630 970	2472 (94.0%) 896 (92.4%) 0.079	158 (6.0%) 74 (7.6%)	2317 (88.1%) 813 (83.8%) **0.001**	313 (11.9%) 157 (16.2%)	1519 (57.8%) 640 (66.0%) **˂0.001**	1111 (42.2%) 330 (34.0%)	1125 (42.8%) 366 (37.7%) **0.006**	1505 (57.2%) 604 (62.3%)
Obesity (BMI ≥ 30)									
No Yes *p*-value *	3378 222	3148 (93.2%) 220 (99.1%) **0.001**	230 (6.8%) 2 (0.9%)	2928 (86.7%) 202 (91.0%) 0.065	450 (13.3%) 20 (9.0%)	2019 (59.8%) 140 (63.1%) 0.332	1359 (40.2%) 82 (36.9%)	1399 (41.4%) 92 (41.4%) 0.994	1979 (58.6%) 130 (58.6%)

* Categorical variables were analyzed by using Chi-squared test and expressed as numbers and percentages. Significant values (*p*-value ˂ 0.05) were presented in bold type.

**Table 2:** Prevalence of daily sugar-sweetened beverage (SSB) consumption among study participants (*n* = 3600) stratified by sociodemographic characteristics and obesity.

Variables	Total N	Total SSBs		Regular Soda		Fruit Drinks		Energy Drinks	
		Yes N (%)	No N (%)	Yes N (%)	No N (%)	Yes N (%)	No N (%)	Yes N (%)	No N (%)
All Participants	3600	1468 (40.8%)	2132 (59.2%)	538 (14.9%)	3062 (85.1%)	140 (3.9%)	3460 (96.1%)	63 (1.8%)	3537 (98.3%)
Nationality									
Saudi Egyptian Yemeni Syrian Jordanian Sudanese Turkish Pakistani Afghan Indian Bangladeshi Filipino *p*-value *	289 289 335 293 280 276 203 306 303 297 350 379	145 (50.2%) 181 (62.6%) 214 (63.9%) 152 (51.9%) 112 (40.0%) 24 (8.7%) 82 (40.4%) 97 (31.7%) 121 (39.9%) 122 (41.1%) 24 (6.9%) 194 (51.2%) **˂0.001**	144 (49.8%) 108 (37.4%) 121 (36.1%) 141 (48.1%) 168 (60.0%) 252 (91.3%) 121 (59.6%) 209 (68.3%) 182 (60.1%) 175 (58.9%) 326 (93.1%) 185 (48.8%)	61 (21.1%) 85 (29.4%) 109 (32.5%) 40 (13.7%) 7 (2.5%) 15 (5.4%) 41 (20.2%) 21 (6.9%) 57 (18.8%) 68 (22.9%) 7 (2.0%) 27 (7.1%) **˂0.001**	228 (78.9%) 204 (70.6%) 226 (67.5%) 253 (86.3%) 273 (97.5%) 261 (94.6%) 162 (79.8%) 285 (93.1%) 246 (81.2%) 229 (77.1%) 343 (98.0%) 352 (92.9%)	12 (4.2%) 32 (11.1%) 39 (11.6%) 14 (4.8%) 6 (2.1%) 0 (0.0%) 4 (2.0%) 0 (0.0%) 1 (0.3%) 4 (1.3%) 0 (0.0%) 28 (7.4%) **˂0.001**	277 (95.8%) 257 (88.9%) 296 (88.4%) 279 (95.2%) 274 (97.9%) 276 (100.0%) 199 (98.0%) 306 (100.0%) 302 (99.7%) 293 (98.7%) 350 (100.0%) 351 (92.6%)	6 (2.1%) 6 (2.1%) 21 (6.3%) 8 (2.7%) 0 (0.0%) 0 (0.0%) 1 (0.5%) 4 (1.3%) 15 (5.0%) 0 (0.0%) 1 (0.3%) 1 (0.3%) **˂0.001**	283 (97.9%) 283 (97.9%) 314 (93.7%) 285 (97.3%) 280 (100.0%) 276 (100.0%) 202 (99.5%) 302 (98.7%) 288 (95.0%) 297 (100.0%) 349 (99.7%) 378 (99.7%)
Residency Period in KSA									
1–5 years 6 years or more *p*-value *	2198 1402	903 (41.1%) 565 (40.3%) 0.641	1295 (58.9%) 837 (59.7%)	314 (14.3%) 224 (16.0%) 0.165	1884 (85.7%) 1178 (84.0%)	86 (3.9%) 54 (3.9%) 0.926	2112 (96.1%) 1348 (96.1%)	39 (1.8%) 24 (1.7%) 0.889	2159 (98.2%) 1378 (98.3%)
Household Type									
Non-family household Family household *p*-value *	2920 680	1157 (39.6%) 311 (45.7%) **0.003**	1763 (60.4%) 369 (54.3%)	428 (14.7%) 110 (16.2%) 0.317	2492 (85.3%) 570 (83.8%)	102 (3.5%) 38 (5.6%) **0.011**	2818 (96.5%) 642 (94.4%)	46 (1.6%) 17 (2.5%) 0.098	2874 (98.4%) 663 (97.5%)
Marital Status									
Single Married *p*-value *	1919 1681	850 (44.3%) 618 (36.8%) **˂0.001**	1069 (55.7%) 1063 (63.2%)	328 (17.1%) 210 (12.5%) **˂0.001**	1591 (82.9%) 1471 (87.5%)	91 (4.7%) 49 (2.9%) **0.005**	1828 (95.3%) 1632 (97.1%)	41 (2.1%) 22 (1.3%) 0.059	1878 (97.9%) 1659 (98.7%)
Education Level									
Low (high school or less) High (college or more) *p*-value *	2284 1316	826 (36.2%) 642 (48.8%) **˂0.001**	1458 (63.8%) 674 (51.2%)	362 (15.8%) 176 (13.4%) **0.045**	1922 (84.2%) 1140 (86.6%)	61 (2.7%) 79 (6.0%) **˂0.001**	2223 (97.3%) 1237 (94.0%)	49 (2.1%) 14 (1.1%) **0.017**	2235 (97.9%) 1302 (98.9%)
Monthly Income									
Low (˂1000 USD) High (≥1000 USD) *p*-value *	2630 970	1066 (40.5%) 402 (41.4%) 0.622	1564 (59.5%) 568 (58.6%)	404 (15.4%) 134 (13.8%) 0.248	2226 (84.6%) 836 (86.2%)	101 (3.8%) 39 (4.0%) 0.804	2529 (96.2%) 931 (96.0%)	50 (1.9%) 13 (1.3%) 0.255	2580 (98.1%) 957 (98.7%)
Obesity (BMI ≥ 30)									
No Yes *p*-value *	3378 222	1366 (40.4%) 102 (45.9%) 0.106	2012 (59.6%) 120 (54.1%)	487 (14.4%) 51 (23.0%) **0.001**	2891 (85.6%) 171 (77.0%)	131 (3.9%) 9 (4.1%) 0.895	3247 (96.1%) 213 (95.9%)	57 (1.7%) 6 (2.7%) 0.264	3321 (98.3%) 216 (97.3%)

* Categorical variables were analyzed by using Chi-squared test and expressed as numbers and percentages. Significant values (*p*-value ˂ 0.05) were presented in bold type.

**Table 3:** Likelihoods of weekly and daily sugar-sweetened beverage (SSB) consumption among study participants for sociodemographic characteristics and obesity.

Variables	Weekly SSB Consumption		Daily SSB Consumption	
	Odds Ratio (95% CI)	*p*-Value *	Odds Ratio (95% CI)	*p*-Value *
Nationality				
Bangladeshi	1.00		1.00	
Saudi Egyptian Yemeni Syrian Jordanian Sudanese Turkish Pakistani Afghan Indian Filipino	6.70 (2.65–16.91) 28.80 (8.58–96.63) 26.66 (9.30–76.44) 5.03 (2.53–10.00) 2.15 (1.20–3.86) 2.34 (1.47–3.71) 14.45 (5.75–36.30) 6.26 (3.29–11.91) 7.99 (4.02–15.88) 9.38 (4.58–19.21) 44.98 (10.40–194.57)	**˂0.001** **˂0.001** **˂0.001** **˂0.001** **0.010** **˂0.001** **˂0.001** **˂0.001** **˂0.001** **˂0.001** **˂0.001**	22.60 (13.01–39.25) 28.22 (17.05–46.70) 29.94 (18.44–48.60) 20.95 (12.56–34.93) 12.98 (7.76–21.73) 1.41 (0.78–2.55) 11.77 (7.06–19.60) 6.69 (4.12–10.85) 9.63 (5.98–15.51) 9.44 (5.86–15.21) 12.83 (7.85–20.95)	**˂0.001** **˂0.001** **˂0.001** **˂0.001** **˂0.001** 0.254 **˂0.001** **˂0.001** **˂0.001** **˂0.001** **˂0.001**
Age (years)	1.06 (1.00–1.12)	0.054	0.96 (0.93–0.98)	**0.001**
Residency Period in KSA				
1–5 years	1.00		1.00	
6 years or more	1.99 (1.38–2.88)	**˂0.001**	1.00 (0.83–1.19)	0.957
Household Type				
Non-family household	1.00		1.00	
Family household	1.34 (0.81–2.21)	0.259	0.80 (0.62–1.02)	0.071
Marital Status				
Single	1.00		1.00	
Married	0.52 (0.37–0.73)	**˂0.001**	1.09 (0.91–1.30)	0.352
Education Level				
Low (high school or less)	1.00		1.00	
High (college or more)	1.45 (0.84–2.49)	0.186	1.12 (0.90–1.39)	0.317
Monthly Income				
Low (˂1000 USD)	1.00		1.00	
High (≥1000 USD)	0.37 (0.23–0.62)	**˂0.001**	0.58 (0.47–0.71)	**˂0.001**
Obesity (BMI ≥ 30)				
No	1.00		1.00	
Yes	4.53 (1.10–18.68)	**0.037**	1.05 (0.78–1.41)	0.734

* Multivariate logistic regression analysis was used. Differences were considered statistically significant at *p*-value < 0.05, and significant values were presented in bold type.

## Data Availability

Data are available from the corresponding author on reasonable request.
